# Dichloridobis(2-chloro­benzyl-κ*C*)[5,6-diphenyl-3-(2-pyrid­yl)-1,2,4-triazine-κ^2^ *N* ^2^,*N* ^3^]tin(IV)

**DOI:** 10.1107/S1600536809019242

**Published:** 2009-05-29

**Authors:** Chui Lian Tan, Kong Mun Lo, Seik Weng Ng

**Affiliations:** a Department of Chemistry, University of Malaya, 50603 Kuala Lumpur, Malaysia

## Abstract

The asymmetric unit of the crystal structure of the title compound, [Sn(C_7_H_6_Cl)_2_Cl_2_(C_20_H_14_N_4_)], contains two independent mol­ecules. Each Sn^IV^ atom is chelated by the bipyridine-like N-heterocycle and exists in a distorted *trans*-C_2_SnCl_2_N_2_ octa­hedral coordination environment. One chloro­benzyl substituent is disordered in each mol­ecule in 0.5:0.5 and 0.778 (2):0.222 (2) ratios.

## Related literature

Several diorganotin dichloride adducts of 2,2′-bipyridine have been reported, *e.g.* the diethyl­tin dichloride, dibutyl­tin dichoride and dibenzyl­tin dichloride adducts, see: Chadha *et al.* (1980 ▶); Gill *et al.* (1999 ▶); Tiekink *et al.* (2000 ▶). The structure of di(2-chloro­benzyl­tin) dichloride has not been reported; for that of di(4-chloro­benzyltin) dichloride, see: Kuang & Feng (2000 ▶). For the direct synthesis of di(chloro­benz­yl)tin dichlorides, see: Sisido *et al.* (1961 ▶).
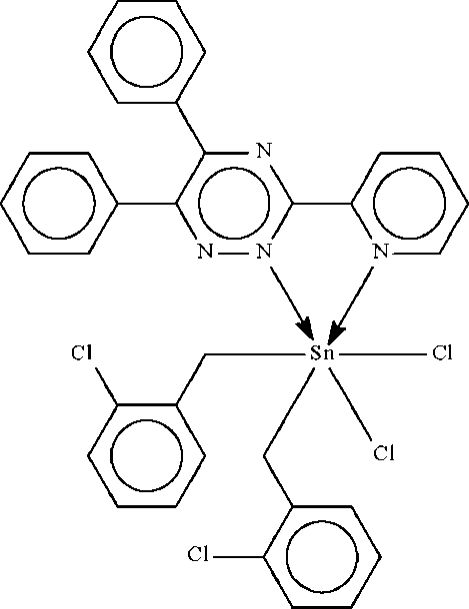


## Experimental

### 

#### Crystal data

[Sn(C_7_H_6_Cl)_2_Cl_2_(C_20_H_14_N_4_)]
*M* *_r_* = 751.08
Monoclinic, 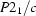

*a* = 31.3421 (4) Å
*b* = 9.9521 (1) Å
*c* = 21.0742 (3) Å
β = 107.044 (1)°
*V* = 6284.75 (14) Å^3^
*Z* = 8
Mo *K*α radiation
μ = 1.19 mm^−1^
*T* = 100 K
0.40 × 0.35 × 0.30 mm

#### Data collection

Bruker SMART APEX diffractometer
Absorption correction: multi-scan (*SADABS*; Sheldrick, 1996 ▶) *T*_min_ = 0.649, *T*_max_ = 0.718
58928 measured reflections
14419 independent reflections
12295 reflections with *I* > 2σ(*I*)
*R*_int_ = 0.031

#### Refinement

*R*[*F* ^2^ > 2σ(*F* ^2^)] = 0.034
*wR*(*F* ^2^) = 0.094
*S* = 1.09
14419 reflections
710 parameters
128 restraints
H-atom parameters constrained
Δρ_max_ = 1.40 e Å^−3^
Δρ_min_ = −0.79 e Å^−3^

### 

Data collection: *APEX2* (Bruker, 2007 ▶); cell refinement: *APEX2*; data reduction: *SAINT* (Bruker, 2007 ▶); program(s) used to solve structure: *SHELXS97* (Sheldrick, 2008 ▶); program(s) used to refine structure: *SHELXL97* (Sheldrick, 2008 ▶); molecular graphics: *X-SEED* (Barbour, 2001 ▶); software used to prepare material for publication: *publCIF* (Westrip, 2009 ▶).

## Supplementary Material

Crystal structure: contains datablocks global, I. DOI: 10.1107/S1600536809019242/xu2527sup1.cif

Structure factors: contains datablocks I. DOI: 10.1107/S1600536809019242/xu2527Isup2.hkl

Additional supplementary materials:  crystallographic information; 3D view; checkCIF report

## Figures and Tables

**Fig. 1.**
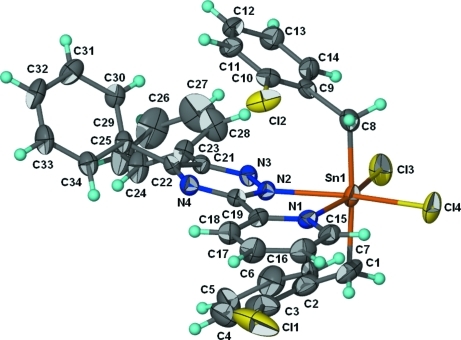
Thermal ellipsoid plot (Barbour, 2001) of one independent molecule of SnCl2(C7H6Cl)2(C20H14N4) at the 70% probability level. Hydrogen atoms are drawn as spheres of arbitrary radius. The disorder is not shown.

**Fig. 2.**
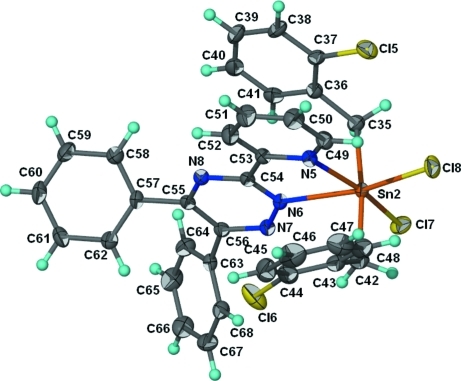
Thermal ellipsoid plot (Barbour, 2001) of second one independent molecule of SnCl2(C7H6Cl)2(C20H14N4) at the 70% probability level. Hydrogen atoms are drawn as spheres of arbitrary radius. The disorder is not shown.

## supplementary crystallographic information

### Experimental

Di(*o*-chlorobenzyl)tin dichloride was synthesized by the reaction of *o*-chlorobenzyl chloride and metallic tin (Sisido *et al.*, 1961). The reactant (1 g, 2.6 mmol) and (colorless) 5,6-diphenyl-3-(2-pyridyl)-1,2,4-triazine (0.83 g, 2.6 mmol) were heated in ethanol for 1 hour. Orange crystals separated from the cool solution after a day.

### Refinement

Parts of the two independent molecules are disordered. As such, the phenyl and phenylene rings were all refined as rigid hexagons of 1.39 Å sides.

The C2/C3/C4/C5/C7/Cl1 and C36/C37/C38/C39/C40/C41/Cl5 groups are disordered over two positions. For the first, the occupancy could not be refined, and was arbitrarily assumed as 50:50. For the second, the occupancy refined to 0.78:0.22. For the disordered groups, the C_methylene_–C_ipso_ distance was restrained to 1.50±0.01 Å and the C–Cl distance to 1.75±0.01 Å. Additionally, the Cl···C_ispo,meta_ distance was restrained to 2.73±0.01 Å. All atoms of each component was restrained to lie on a plane, with maximum deviation of ±0.01 Å. The temperature factors of the primed carbon atoms were set to those of the unprimed ones. The anisotropic temperature factors of all disordered atoms were restrained to be nearly isotropic.

Hydrogen atoms were placed at calculated positions (C–H 0.95 Å) and were treated as riding on their parent atoms, with *U*(H) set to 1.2 times *U*_eq_(C).

The final difference Fourier map had a large peak at 0.5 Å from C7' but was otherwise featureless.

### Crystal data

**Table:**

[Sn(C_7_H_6_Cl)_2_Cl_2_(C_20_H_14_N_4_)]	*F*(000) = 3008
*M**_r_* = 751.08	*D*_x_ = 1.588 Mg m^−^^3^
Monoclinic, *P*2_1_/*c*	Mo *K*α radiation, λ = 0.71073 Å
Hall symbol: -P 2ybc	Cell parameters from 9969 reflections
*a* = 31.3421 (4) Å	θ = 2.3–28.2°
*b* = 9.9521 (1) Å	µ = 1.19 mm^−^^1^
*c* = 21.0742 (3) Å	*T* = 100 K
β = 107.044 (1)°	Block, orange
*V* = 6284.75 (14) Å^3^	0.40 × 0.35 × 0.30 mm
*Z* = 8	

### Data collection

**Table:**

Bruker SMART APEX diffractometer	14419 independent reflections
Radiation source: fine-focus sealed tube	12295 reflections with *I* > 2σ(*I*)
graphite	*R*_int_ = 0.031
ω scans	θ_max_ = 27.5°, θ_min_ = 0.7°
Absorption correction: multi-scan (*SADABS*; Sheldrick, 1996)	*h* = −40→40
*T*_min_ = 0.649, *T*_max_ = 0.718	*k* = −12→12
58928 measured reflections	*l* = −27→27

### Refinement

**Table:**

Refinement on *F*^2^	Primary atom site location: structure-invariant direct methods
Least-squares matrix: full	Secondary atom site location: difference Fourier map
*R*[*F*^2^ > 2σ(*F*^2^)] = 0.034	Hydrogen site location: inferred from neighbouring sites
*wR*(*F*^2^) = 0.094	H-atom parameters constrained
*S* = 1.09	*w* = 1/[σ^2^(*F*_o_^2^) + (0.05*P*)^2^ + 5*P*] where *P* = (*F*_o_^2^ + 2*F*_c_^2^)/3
14419 reflections	(Δ/σ)_max_ = 0.001
710 parameters	Δρ_max_ = 1.40 e Å^−^^3^
128 restraints	Δρ_min_ = −0.79 e Å^−^^3^

### Fractional atomic coordinates and isotropic or equivalent isotropic displacement parameters (Å^2^)

**Table:**

	*x*	*y*	*z*	*U*_iso_*/*U*_eq_	Occ. (<1)
Sn1	0.337678 (7)	0.85029 (2)	0.245879 (10)	0.02394 (6)	
Sn2	0.115732 (6)	0.468724 (17)	0.150816 (8)	0.01496 (5)	
Cl1	0.4189 (2)	0.5202 (6)	0.3582 (3)	0.0792 (14)	0.50
Cl1'	0.4120 (2)	0.5487 (7)	0.3809 (3)	0.121 (3)	0.50
Cl2	0.25217 (3)	0.57651 (10)	0.09862 (4)	0.0409 (2)	
Cl3	0.37108 (3)	1.05914 (8)	0.21457 (5)	0.0419 (2)	
Cl4	0.28992 (3)	0.95136 (9)	0.30908 (4)	0.0401 (2)	
Cl5	0.09515 (3)	0.25073 (10)	−0.02284 (4)	0.0269 (3)	0.778 (2)
Cl5'	0.21414 (12)	0.1384 (4)	0.19930 (17)	0.0424 (12)	0.222 (2)
Cl6	0.06462 (3)	0.37633 (10)	0.31987 (4)	0.0399 (2)	
Cl7	0.17737 (2)	0.62965 (7)	0.19768 (3)	0.02471 (14)	
Cl8	0.07577 (2)	0.58752 (7)	0.04745 (3)	0.02630 (15)	
N1	0.31418 (8)	0.6252 (2)	0.26478 (11)	0.0232 (5)	
N2	0.37008 (8)	0.6853 (2)	0.19349 (11)	0.0215 (5)	
N3	0.39714 (8)	0.7224 (2)	0.15831 (12)	0.0222 (5)	
N4	0.36586 (8)	0.4630 (2)	0.15341 (12)	0.0236 (5)	
N5	0.06326 (7)	0.2843 (2)	0.12295 (10)	0.0170 (4)	
N6	0.13203 (7)	0.2986 (2)	0.23490 (10)	0.0164 (4)	
N7	0.16371 (7)	0.3167 (2)	0.29263 (10)	0.0168 (4)	
N8	0.12132 (7)	0.0720 (2)	0.26044 (10)	0.0176 (4)	
C1	0.39403 (11)	0.8287 (4)	0.33495 (16)	0.0400 (8)	
H1A	0.4007	0.9178	0.3565	0.048*	0.50
H1B	0.3850	0.7684	0.3661	0.048*	0.50
H1C	0.4008	0.9181	0.3561	0.048*	0.50
H1D	0.3848	0.7696	0.3664	0.048*	0.50
C2	0.4366 (4)	0.7738 (8)	0.3242 (8)	0.0403 (8)	0.50
C3	0.4495 (2)	0.6396 (7)	0.3311 (3)	0.0553 (16)	0.50
C4	0.4881 (3)	0.5992 (6)	0.3167 (5)	0.054 (2)	0.50
H4	0.4969	0.5075	0.3214	0.065*	0.50
C5	0.5138 (3)	0.6929 (8)	0.2953 (6)	0.061 (3)	0.50
H5	0.5402	0.6653	0.2855	0.073*	0.50
C6	0.5009 (3)	0.8270 (6)	0.2884 (6)	0.053 (2)	0.50
H6	0.5185	0.8911	0.2738	0.063*	0.50
C7	0.4623 (4)	0.8675 (6)	0.3028 (9)	0.0396 (15)	0.50
H7	0.4535	0.9591	0.2981	0.048*	0.50
C2'	0.4366 (4)	0.7723 (9)	0.3252 (8)	0.0403 (8)	0.50
C3'	0.4454 (2)	0.6389 (8)	0.3439 (3)	0.0553 (16)	0.50
C4'	0.4823 (3)	0.5748 (7)	0.3336 (5)	0.054 (2)	0.50
H4'	0.4883	0.4836	0.3464	0.065*	0.50
C5'	0.5102 (3)	0.6441 (8)	0.3046 (6)	0.061 (3)	0.50
H5'	0.5354	0.6004	0.2976	0.073*	0.50
C6'	0.5014 (3)	0.7776 (7)	0.2859 (6)	0.053 (2)	0.50
H6'	0.5205	0.8250	0.2661	0.063*	0.50
C7'	0.4645 (4)	0.8417 (7)	0.2962 (9)	0.0396 (15)	0.50
H7'	0.4585	0.9329	0.2834	0.048*	0.50
C8	0.28281 (11)	0.8601 (3)	0.15484 (15)	0.0295 (7)	
H8A	0.2583	0.8034	0.1607	0.035*	
H8B	0.2718	0.9538	0.1491	0.035*	
C9	0.29205 (7)	0.81706 (19)	0.09063 (7)	0.0249 (6)	
C10	0.27806 (6)	0.69252 (17)	0.06227 (9)	0.0260 (6)	
C11	0.28485 (7)	0.65793 (15)	0.00206 (9)	0.0300 (7)	
H11	0.2753	0.5728	−0.0173	0.036*	
C12	0.30564 (7)	0.74788 (19)	−0.02978 (7)	0.0291 (6)	
H12	0.3103	0.7242	−0.0709	0.035*	
C13	0.31963 (7)	0.87241 (18)	−0.00141 (9)	0.0330 (7)	
H13	0.3338	0.9339	−0.0232	0.040*	
C14	0.31283 (7)	0.90701 (15)	0.05880 (9)	0.0305 (7)	
H14	0.3224	0.9921	0.0782	0.037*	
C15	0.28733 (10)	0.5980 (3)	0.30197 (14)	0.0278 (6)	
H15	0.2750	0.6707	0.3200	0.033*	
C16	0.27668 (11)	0.4680 (4)	0.31529 (16)	0.0352 (7)	
H16	0.2579	0.4520	0.3426	0.042*	
C17	0.29383 (12)	0.3624 (4)	0.28822 (17)	0.0367 (8)	
H17	0.2873	0.2724	0.2973	0.044*	
C18	0.32066 (11)	0.3883 (3)	0.24760 (15)	0.0311 (7)	
H18	0.3323	0.3170	0.2276	0.037*	
C19	0.33003 (10)	0.5215 (3)	0.23706 (14)	0.0240 (6)	
C20	0.35760 (9)	0.5572 (3)	0.19343 (13)	0.0213 (6)	
C21	0.40822 (9)	0.6320 (3)	0.11932 (14)	0.0211 (5)	
C22	0.38876 (9)	0.5015 (3)	0.11261 (14)	0.0207 (5)	
C23	0.44094 (6)	0.6784 (2)	0.08617 (11)	0.0254 (6)	
C24	0.47436 (7)	0.59355 (18)	0.07872 (13)	0.0377 (8)	
H24	0.4762	0.5039	0.0948	0.045*	
C25	0.50510 (7)	0.6399 (2)	0.04782 (14)	0.0536 (11)	
H25	0.5279	0.5818	0.0427	0.064*	
C26	0.50240 (8)	0.7711 (3)	0.02436 (15)	0.0614 (13)	
H26	0.5234	0.8027	0.0032	0.074*	
C27	0.46898 (9)	0.8560 (2)	0.03180 (15)	0.0652 (13)	
H27	0.4671	0.9456	0.0158	0.078*	
C28	0.43825 (7)	0.8096 (2)	0.06271 (14)	0.0450 (9)	
H28	0.4154	0.8677	0.0678	0.054*	
C29	0.38923 (7)	0.40531 (16)	0.05922 (8)	0.0221 (6)	
C30	0.38156 (7)	0.44915 (14)	−0.00579 (9)	0.0264 (6)	
H30	0.3798	0.5426	−0.0154	0.032*	
C31	0.37653 (7)	0.3563 (2)	−0.05672 (7)	0.0345 (7)	
H31	0.3713	0.3862	−0.1011	0.041*	
C32	0.37917 (8)	0.21954 (18)	−0.04264 (9)	0.0392 (8)	
H32	0.3757	0.1561	−0.0774	0.047*	
C33	0.38684 (8)	0.17569 (13)	0.02237 (10)	0.0363 (8)	
H33	0.3886	0.0822	0.0320	0.044*	
C34	0.39187 (7)	0.26858 (17)	0.07330 (7)	0.0280 (6)	
H34	0.3971	0.2386	0.1177	0.034*	
C35	0.16054 (9)	0.3571 (3)	0.10712 (14)	0.0204 (5)	
H35A	0.1576	0.3964	0.0629	0.025*	0.778 (2)
H35B	0.1915	0.3742	0.1349	0.025*	0.778 (2)
H35C	0.1622	0.4056	0.0669	0.025*	0.222 (2)
H35D	0.1908	0.3581	0.1392	0.025*	0.222 (2)
C36	0.15491 (9)	0.20565 (15)	0.09827 (12)	0.0188 (6)	0.778 (2)
C37	0.12506 (7)	0.1509 (2)	0.04190 (10)	0.0185 (7)	0.778 (2)
C38	0.11928 (7)	0.0124 (2)	0.03626 (10)	0.0228 (7)	0.778 (2)
H38	0.0989	−0.0250	−0.0023	0.027*	0.778 (2)
C39	0.14334 (9)	−0.07120 (15)	0.08700 (13)	0.0270 (9)	0.778 (2)
H39	0.1394	−0.1658	0.0831	0.032*	0.778 (2)
C40	0.17319 (8)	−0.0164 (2)	0.14337 (10)	0.0263 (8)	0.778 (2)
H40	0.1896	−0.0736	0.1780	0.032*	0.778 (2)
C41	0.17897 (8)	0.1220 (2)	0.14900 (10)	0.0237 (8)	0.778 (2)
H41	0.1994	0.1594	0.1875	0.028*	0.778 (2)
C36'	0.1501 (3)	0.2140 (5)	0.0872 (4)	0.0188 (6)	0.22
C37'	0.17185 (18)	0.1093 (7)	0.1275 (3)	0.0185 (7)	0.22
C38'	0.1598 (3)	−0.0230 (6)	0.1098 (4)	0.0228 (7)	0.22
H38'	0.1747	−0.0945	0.1374	0.027*	0.222 (2)
C39'	0.1261 (3)	−0.0506 (6)	0.0518 (4)	0.0270 (9)	0.22
H39'	0.1179	−0.1410	0.0397	0.032*	0.222 (2)
C40'	0.1043 (2)	0.0541 (9)	0.0115 (3)	0.0263 (8)	0.22
H40'	0.0813	0.0352	−0.0282	0.032*	0.222 (2)
C41'	0.1163 (3)	0.1864 (7)	0.0292 (4)	0.0237 (8)	0.22
H41'	0.1015	0.2579	0.0016	0.028*	0.222 (2)
C42	0.07750 (9)	0.5679 (3)	0.20853 (14)	0.0221 (6)	
H42A	0.0950	0.5653	0.2560	0.026*	
H42B	0.0735	0.6635	0.1950	0.026*	
C43	0.03171 (5)	0.50634 (19)	0.20108 (9)	0.0228 (6)	
C44	0.02529 (6)	0.4103 (2)	0.24530 (8)	0.0274 (6)	
C45	−0.01549 (7)	0.34462 (19)	0.23260 (10)	0.0335 (7)	
H45	−0.0199	0.2790	0.2628	0.040*	
C46	−0.04984 (5)	0.3749 (2)	0.17567 (11)	0.0378 (8)	
H46	−0.0777	0.3300	0.1670	0.045*	
C47	−0.04341 (5)	0.4709 (2)	0.13145 (8)	0.0339 (7)	
H47	−0.0669	0.4916	0.0925	0.041*	
C48	−0.00264 (6)	0.53663 (18)	0.14415 (8)	0.0281 (6)	
H48	0.0018	0.6023	0.1139	0.034*	
C49	0.02739 (9)	0.2825 (3)	0.06977 (13)	0.0200 (5)	
H49	0.0204	0.3607	0.0428	0.024*	
C50	−0.00022 (10)	0.1705 (3)	0.05223 (14)	0.0252 (6)	
H50	−0.0257	0.1729	0.0144	0.030*	
C51	0.01004 (10)	0.0563 (3)	0.09070 (15)	0.0270 (6)	
H51	−0.0081	−0.0217	0.0793	0.032*	
C52	0.04732 (10)	0.0565 (3)	0.14657 (14)	0.0248 (6)	
H52	0.0551	−0.0209	0.1739	0.030*	
C53	0.07269 (9)	0.1726 (3)	0.16110 (12)	0.0173 (5)	
C54	0.11097 (9)	0.1814 (3)	0.22158 (12)	0.0164 (5)	
C55	0.15296 (9)	0.0868 (3)	0.31798 (12)	0.0168 (5)	
C56	0.17343 (8)	0.2149 (3)	0.33523 (12)	0.0164 (5)	
C57	0.16356 (6)	−0.03552 (13)	0.35977 (7)	0.0171 (5)	
C58	0.16988 (7)	−0.15594 (16)	0.33036 (6)	0.0220 (6)	
H58	0.1708	−0.1569	0.2857	0.026*	
C59	0.17487 (7)	−0.27500 (13)	0.36628 (8)	0.0262 (6)	
H59	0.1792	−0.3573	0.3462	0.031*	
C60	0.17353 (7)	−0.27362 (13)	0.43162 (8)	0.0262 (6)	
H60	0.1769	−0.3550	0.4562	0.031*	
C61	0.16720 (7)	−0.15320 (17)	0.46104 (6)	0.0244 (6)	
H61	0.1663	−0.1523	0.5057	0.029*	
C62	0.16222 (6)	−0.03414 (13)	0.42512 (8)	0.0200 (5)	
H62	0.1579	0.0482	0.4452	0.024*	
C63	0.20575 (5)	0.24691 (17)	0.40048 (6)	0.0162 (5)	
C64	0.24460 (6)	0.17252 (16)	0.42544 (8)	0.0233 (6)	
H64	0.2521	0.1041	0.3991	0.028*	
C65	0.27250 (5)	0.19829 (18)	0.48887 (9)	0.0283 (6)	
H65	0.2990	0.1474	0.5059	0.034*	
C66	0.26154 (6)	0.29845 (19)	0.52734 (7)	0.0282 (6)	
H66	0.2806	0.3161	0.5707	0.034*	
C67	0.22269 (6)	0.37284 (17)	0.50239 (8)	0.0248 (6)	
H67	0.2152	0.4413	0.5287	0.030*	
C68	0.19479 (5)	0.34707 (16)	0.43896 (8)	0.0195 (5)	
H68	0.1682	0.3979	0.4219	0.023*	

### Atomic displacement parameters (Å^2^)

**Table:**

	*U*^11^	*U*^22^	*U*^33^	*U*^12^	*U*^13^	*U*^23^
Sn1	0.02993 (11)	0.02181 (11)	0.02394 (10)	0.00041 (8)	0.01392 (8)	−0.00433 (7)
Sn2	0.01800 (9)	0.01282 (9)	0.01382 (8)	0.00062 (6)	0.00430 (6)	0.00134 (6)
Cl1	0.0621 (18)	0.0562 (18)	0.090 (4)	−0.0076 (14)	−0.023 (2)	0.037 (2)
Cl1'	0.108 (4)	0.113 (5)	0.095 (4)	−0.062 (4)	−0.045 (3)	0.071 (3)
Cl2	0.0391 (4)	0.0525 (5)	0.0281 (4)	−0.0224 (4)	0.0049 (3)	0.0053 (4)
Cl3	0.0528 (5)	0.0230 (4)	0.0562 (5)	−0.0057 (4)	0.0257 (4)	−0.0024 (4)
Cl4	0.0532 (5)	0.0366 (4)	0.0424 (4)	0.0019 (4)	0.0325 (4)	−0.0098 (3)
Cl5	0.0294 (5)	0.0326 (5)	0.0181 (4)	0.0024 (4)	0.0057 (3)	0.0001 (3)
Cl5'	0.0318 (19)	0.064 (3)	0.0287 (18)	0.0202 (17)	0.0038 (14)	−0.0070 (16)
Cl6	0.0356 (4)	0.0606 (6)	0.0264 (4)	0.0116 (4)	0.0134 (3)	0.0139 (4)
Cl7	0.0258 (3)	0.0202 (3)	0.0283 (3)	−0.0052 (3)	0.0081 (3)	−0.0069 (3)
Cl8	0.0308 (4)	0.0243 (3)	0.0205 (3)	0.0025 (3)	0.0024 (3)	0.0084 (3)
N1	0.0251 (12)	0.0269 (13)	0.0190 (11)	0.0013 (10)	0.0085 (10)	0.0016 (9)
N2	0.0209 (12)	0.0234 (12)	0.0209 (11)	0.0007 (9)	0.0071 (9)	−0.0021 (9)
N3	0.0191 (11)	0.0222 (12)	0.0267 (12)	0.0003 (9)	0.0085 (10)	−0.0020 (9)
N4	0.0273 (13)	0.0208 (12)	0.0245 (12)	0.0009 (10)	0.0103 (10)	0.0013 (9)
N5	0.0182 (11)	0.0174 (11)	0.0161 (10)	−0.0003 (9)	0.0061 (8)	0.0013 (8)
N6	0.0196 (11)	0.0140 (10)	0.0160 (10)	0.0002 (8)	0.0057 (8)	0.0012 (8)
N7	0.0181 (11)	0.0164 (11)	0.0160 (10)	0.0011 (9)	0.0053 (8)	0.0014 (8)
N8	0.0215 (11)	0.0159 (11)	0.0152 (10)	0.0000 (9)	0.0051 (9)	0.0001 (8)
C1	0.0393 (19)	0.056 (2)	0.0238 (15)	−0.0074 (17)	0.0079 (14)	−0.0131 (15)
C2	0.047 (2)	0.040 (2)	0.0267 (16)	−0.0051 (16)	0.0001 (15)	−0.0064 (14)
C3	0.059 (3)	0.050 (3)	0.041 (3)	−0.008 (2)	−0.011 (2)	0.003 (2)
C4	0.052 (3)	0.045 (3)	0.051 (5)	0.014 (3)	−0.006 (3)	−0.001 (3)
C5	0.059 (3)	0.048 (7)	0.063 (4)	0.028 (4)	−0.003 (3)	−0.010 (5)
C6	0.057 (3)	0.045 (6)	0.054 (3)	−0.007 (4)	0.014 (2)	−0.010 (5)
C7	0.047 (2)	0.023 (3)	0.039 (3)	0.015 (2)	−0.0008 (19)	−0.011 (3)
C2'	0.047 (2)	0.040 (2)	0.0267 (16)	−0.0051 (16)	0.0001 (15)	−0.0064 (14)
C3'	0.059 (3)	0.050 (3)	0.041 (3)	−0.008 (2)	−0.011 (2)	0.003 (2)
C4'	0.052 (3)	0.045 (3)	0.051 (5)	0.014 (3)	−0.006 (3)	−0.001 (3)
C5'	0.059 (3)	0.048 (7)	0.063 (4)	0.028 (4)	−0.003 (3)	−0.010 (5)
C6'	0.057 (3)	0.045 (6)	0.054 (3)	−0.007 (4)	0.014 (2)	−0.010 (5)
C7'	0.047 (2)	0.023 (3)	0.039 (3)	0.015 (2)	−0.0008 (19)	−0.011 (3)
C8	0.0294 (16)	0.0317 (17)	0.0301 (16)	0.0083 (13)	0.0129 (13)	0.0022 (12)
C9	0.0207 (14)	0.0276 (15)	0.0264 (14)	0.0080 (11)	0.0069 (11)	0.0022 (12)
C10	0.0202 (14)	0.0308 (16)	0.0258 (14)	−0.0019 (12)	0.0051 (11)	0.0056 (12)
C11	0.0349 (17)	0.0286 (16)	0.0236 (14)	−0.0037 (13)	0.0038 (13)	−0.0022 (12)
C12	0.0323 (16)	0.0333 (17)	0.0217 (14)	0.0028 (13)	0.0079 (12)	−0.0016 (12)
C13	0.0394 (18)	0.0321 (17)	0.0299 (16)	−0.0022 (14)	0.0138 (14)	0.0033 (13)
C14	0.0391 (18)	0.0250 (16)	0.0296 (15)	0.0023 (13)	0.0136 (14)	0.0002 (12)
C15	0.0268 (15)	0.0370 (17)	0.0217 (14)	0.0002 (13)	0.0102 (12)	−0.0003 (12)
C16	0.0359 (18)	0.043 (2)	0.0320 (16)	−0.0037 (15)	0.0187 (14)	0.0044 (14)
C17	0.045 (2)	0.0334 (18)	0.0356 (17)	−0.0040 (15)	0.0186 (16)	0.0055 (14)
C18	0.0402 (18)	0.0276 (16)	0.0290 (15)	0.0016 (14)	0.0157 (14)	0.0040 (12)
C19	0.0273 (14)	0.0262 (15)	0.0192 (13)	0.0007 (12)	0.0079 (11)	0.0011 (11)
C20	0.0210 (13)	0.0219 (14)	0.0202 (13)	0.0016 (11)	0.0048 (11)	0.0006 (10)
C21	0.0181 (13)	0.0223 (14)	0.0230 (13)	0.0011 (11)	0.0059 (11)	−0.0009 (11)
C22	0.0199 (13)	0.0183 (13)	0.0245 (13)	0.0037 (10)	0.0073 (11)	0.0016 (10)
C23	0.0209 (14)	0.0270 (15)	0.0314 (15)	−0.0066 (12)	0.0121 (12)	−0.0067 (12)
C24	0.0296 (17)	0.0344 (18)	0.056 (2)	−0.0068 (14)	0.0237 (16)	−0.0124 (16)
C25	0.038 (2)	0.056 (3)	0.079 (3)	−0.0167 (18)	0.038 (2)	−0.032 (2)
C26	0.049 (2)	0.071 (3)	0.082 (3)	−0.028 (2)	0.047 (2)	−0.015 (2)
C27	0.057 (3)	0.057 (3)	0.093 (4)	−0.016 (2)	0.041 (3)	0.020 (2)
C28	0.0377 (19)	0.037 (2)	0.069 (3)	−0.0043 (16)	0.0299 (19)	0.0091 (18)
C29	0.0188 (13)	0.0210 (14)	0.0282 (14)	−0.0018 (11)	0.0096 (11)	−0.0051 (11)
C30	0.0265 (15)	0.0252 (15)	0.0300 (15)	−0.0031 (12)	0.0124 (12)	−0.0026 (12)
C31	0.0361 (18)	0.043 (2)	0.0289 (16)	−0.0083 (15)	0.0164 (14)	−0.0075 (14)
C32	0.045 (2)	0.0374 (19)	0.0412 (19)	−0.0057 (15)	0.0211 (16)	−0.0182 (15)
C33	0.0418 (19)	0.0210 (16)	0.049 (2)	−0.0002 (14)	0.0185 (16)	−0.0070 (14)
C34	0.0273 (15)	0.0219 (15)	0.0360 (16)	−0.0006 (12)	0.0109 (13)	−0.0018 (12)
C35	0.0232 (14)	0.0171 (13)	0.0236 (13)	−0.0013 (10)	0.0109 (11)	−0.0016 (10)
C36	0.0216 (15)	0.0170 (14)	0.0213 (16)	0.0001 (11)	0.0118 (13)	0.0002 (11)
C37	0.0196 (17)	0.0166 (17)	0.0228 (17)	0.0021 (13)	0.0116 (14)	−0.0018 (13)
C38	0.0257 (18)	0.0137 (17)	0.0322 (19)	−0.0022 (13)	0.0138 (16)	−0.0054 (14)
C39	0.035 (2)	0.0134 (17)	0.040 (2)	−0.0002 (15)	0.0228 (19)	−0.0004 (16)
C40	0.034 (2)	0.0224 (18)	0.0302 (19)	0.0082 (15)	0.0206 (17)	0.0036 (15)
C41	0.0279 (19)	0.0250 (18)	0.0219 (17)	0.0034 (15)	0.0131 (15)	−0.0021 (14)
C36'	0.0216 (15)	0.0170 (14)	0.0213 (16)	0.0001 (11)	0.0118 (13)	0.0002 (11)
C37'	0.0196 (17)	0.0166 (17)	0.0228 (17)	0.0021 (13)	0.0116 (14)	−0.0018 (13)
C38'	0.0257 (18)	0.0137 (17)	0.0322 (19)	−0.0022 (13)	0.0138 (16)	−0.0054 (14)
C39'	0.035 (2)	0.0134 (17)	0.040 (2)	−0.0002 (15)	0.0228 (19)	−0.0004 (16)
C40'	0.034 (2)	0.0224 (18)	0.0302 (19)	0.0082 (15)	0.0206 (17)	0.0036 (15)
C41'	0.0279 (19)	0.0250 (18)	0.0219 (17)	0.0034 (15)	0.0131 (15)	−0.0021 (14)
C42	0.0238 (14)	0.0210 (14)	0.0226 (13)	0.0026 (11)	0.0086 (11)	−0.0032 (11)
C43	0.0273 (15)	0.0194 (14)	0.0247 (14)	0.0017 (11)	0.0125 (12)	−0.0022 (11)
C44	0.0295 (15)	0.0295 (16)	0.0268 (14)	0.0082 (13)	0.0136 (12)	0.0036 (12)
C45	0.0345 (17)	0.0289 (17)	0.0450 (19)	0.0005 (13)	0.0239 (15)	0.0052 (14)
C46	0.0262 (16)	0.042 (2)	0.050 (2)	−0.0025 (14)	0.0178 (15)	−0.0062 (16)
C47	0.0238 (15)	0.045 (2)	0.0325 (16)	0.0075 (14)	0.0073 (13)	−0.0024 (14)
C48	0.0282 (15)	0.0286 (16)	0.0292 (15)	0.0058 (12)	0.0108 (13)	0.0003 (12)
C49	0.0200 (13)	0.0225 (14)	0.0163 (12)	0.0018 (11)	0.0033 (10)	0.0026 (10)
C50	0.0212 (14)	0.0272 (15)	0.0236 (14)	−0.0007 (11)	0.0009 (11)	−0.0009 (11)
C51	0.0265 (15)	0.0244 (15)	0.0277 (15)	−0.0076 (12)	0.0041 (12)	−0.0010 (12)
C52	0.0288 (15)	0.0215 (14)	0.0221 (13)	−0.0025 (12)	0.0044 (12)	0.0030 (11)
C53	0.0202 (13)	0.0170 (13)	0.0155 (11)	0.0010 (10)	0.0063 (10)	0.0009 (9)
C54	0.0204 (13)	0.0156 (12)	0.0153 (11)	0.0011 (10)	0.0085 (10)	0.0006 (9)
C55	0.0202 (13)	0.0168 (13)	0.0161 (11)	0.0021 (10)	0.0094 (10)	0.0008 (10)
C56	0.0181 (12)	0.0169 (12)	0.0156 (11)	0.0035 (10)	0.0072 (10)	0.0009 (9)
C57	0.0175 (12)	0.0147 (12)	0.0182 (12)	0.0006 (10)	0.0042 (10)	0.0040 (9)
C58	0.0278 (14)	0.0193 (14)	0.0185 (13)	0.0016 (11)	0.0060 (11)	0.0003 (10)
C59	0.0333 (16)	0.0150 (13)	0.0292 (15)	0.0019 (11)	0.0075 (12)	−0.0005 (11)
C60	0.0305 (15)	0.0180 (14)	0.0272 (14)	0.0016 (11)	0.0041 (12)	0.0086 (11)
C61	0.0289 (15)	0.0263 (15)	0.0178 (13)	−0.0008 (12)	0.0068 (11)	0.0061 (11)
C62	0.0216 (13)	0.0186 (13)	0.0196 (12)	0.0016 (10)	0.0055 (10)	0.0012 (10)
C63	0.0164 (12)	0.0156 (12)	0.0165 (11)	−0.0007 (10)	0.0045 (10)	0.0030 (9)
C64	0.0225 (14)	0.0206 (14)	0.0266 (14)	0.0028 (11)	0.0068 (11)	−0.0020 (11)
C65	0.0189 (14)	0.0283 (16)	0.0317 (15)	0.0056 (12)	−0.0018 (12)	0.0022 (12)
C66	0.0272 (15)	0.0280 (16)	0.0225 (14)	−0.0018 (12)	−0.0033 (12)	−0.0017 (12)
C67	0.0280 (15)	0.0238 (14)	0.0213 (13)	−0.0017 (12)	0.0050 (12)	−0.0048 (11)
C68	0.0186 (13)	0.0187 (13)	0.0198 (12)	0.0013 (10)	0.0037 (10)	0.0014 (10)

### Geometric parameters (Å, °)

**Table:**

Sn1—C8	2.171 (3)	C26—C27	1.3900
Sn1—C1	2.179 (3)	C26—H26	0.9500
Sn1—N2	2.367 (2)	C27—C28	1.3900
Sn1—N1	2.427 (2)	C27—H27	0.9500
Sn1—Cl4	2.4889 (8)	C28—H28	0.9500
Sn1—Cl3	2.5014 (9)	C29—C30	1.3900
Sn2—C42	2.178 (3)	C29—C34	1.3900
Sn2—C35	2.194 (3)	C30—C31	1.3900
Sn2—N6	2.395 (2)	C30—H30	0.9500
Sn2—N5	2.419 (2)	C31—C32	1.3900
Sn2—Cl8	2.4722 (7)	C31—H31	0.9500
Sn2—Cl7	2.4808 (7)	C32—C33	1.3900
Cl1—C3	1.728 (5)	C32—H32	0.9500
Cl1'—C3'	1.728 (5)	C33—C34	1.3900
Cl2—C10	1.7156 (15)	C33—H33	0.9500
Cl5—C37	1.7258 (19)	C34—H34	0.9500
Cl5'—C37'	1.719 (5)	C35—C36'	1.493 (5)
Cl6—C44	1.7228 (16)	C35—C36	1.522 (3)
N1—C15	1.335 (4)	C35—H35A	0.9900
N1—C19	1.350 (4)	C35—H35B	0.9900
N2—N3	1.332 (3)	C35—H35C	0.9899
N2—C20	1.333 (4)	C35—H35D	0.9899
N3—C21	1.332 (4)	C36—C37	1.3900
N4—C22	1.328 (4)	C36—C41	1.3900
N4—C20	1.336 (4)	C37—C38	1.3900
N5—C49	1.335 (3)	C38—C39	1.3900
N5—C53	1.352 (3)	C38—H38	0.9500
N6—C54	1.329 (3)	C39—C40	1.3900
N6—N7	1.338 (3)	C39—H39	0.9500
N7—C56	1.329 (3)	C40—C41	1.3900
N8—C55	1.331 (3)	C40—H40	0.9500
N8—C54	1.344 (3)	C41—H41	0.9500
C1—C2'	1.515 (7)	C36'—C37'	1.3900
C1—C2	1.521 (7)	C36'—C41'	1.3900
C1—H1A	0.9900	C37'—C38'	1.3900
C1—H1B	0.9900	C38'—C39'	1.3900
C1—H1C	0.9900	C38'—H38'	0.9500
C1—H1D	0.9900	C39'—C40'	1.3900
C2—C3	1.3900	C39'—H39'	0.9500
C2—C7	1.3900	C40'—C41'	1.3900
C3—C4	1.3900	C40'—H40'	0.9500
C4—C5	1.3900	C41'—H41'	0.9500
C4—H4	0.9500	C42—C43	1.526 (3)
C5—C6	1.3900	C42—H42A	0.9900
C5—H5	0.9500	C42—H42B	0.9900
C6—C7	1.3900	C43—C44	1.3900
C6—H6	0.9500	C43—C48	1.3900
C7—H7	0.9500	C44—C45	1.3900
C2'—C3'	1.3900	C45—C46	1.3900
C2'—C7'	1.3900	C45—H45	0.9500
C3'—C4'	1.3900	C46—C47	1.3900
C4'—C5'	1.3900	C46—H46	0.9500
C4'—H4'	0.9500	C47—C48	1.3900
C5'—C6'	1.3900	C47—H47	0.9500
C5'—H5'	0.9500	C48—H48	0.9500
C6'—C7'	1.3900	C49—C50	1.393 (4)
C6'—H6'	0.9500	C49—H49	0.9500
C7'—H7'	0.9500	C50—C51	1.379 (4)
C8—C9	1.526 (3)	C50—H50	0.9500
C8—H8A	0.9900	C51—C52	1.395 (4)
C8—H8B	0.9900	C51—H51	0.9500
C9—C10	1.3900	C52—C53	1.385 (4)
C9—C14	1.3900	C52—H52	0.9500
C10—C11	1.3900	C53—C54	1.475 (4)
C11—C12	1.3900	C55—C56	1.426 (4)
C11—H11	0.9500	C55—C57	1.482 (3)
C12—C13	1.3900	C56—C63	1.484 (3)
C12—H12	0.9500	C57—C58	1.3900
C13—C14	1.3900	C57—C62	1.3900
C13—H13	0.9500	C58—C59	1.3900
C14—H14	0.9500	C58—H58	0.9500
C15—C16	1.385 (5)	C59—C60	1.3900
C15—H15	0.9500	C59—H59	0.9500
C16—C17	1.378 (5)	C60—C61	1.3900
C16—H16	0.9500	C60—H60	0.9500
C17—C18	1.389 (4)	C61—C62	1.3900
C17—H17	0.9500	C61—H61	0.9500
C18—C19	1.390 (4)	C62—H62	0.9500
C18—H18	0.9500	C63—C64	1.3900
C19—C20	1.478 (4)	C63—C68	1.3900
C21—C22	1.425 (4)	C64—C65	1.3900
C21—C23	1.474 (3)	C64—H64	0.9500
C22—C29	1.480 (3)	C65—C66	1.3900
C23—C24	1.3900	C65—H65	0.9500
C23—C28	1.3900	C66—C67	1.3900
C24—C25	1.3900	C66—H66	0.9500
C24—H24	0.9500	C67—C68	1.3900
C25—C26	1.3900	C67—H67	0.9500
C25—H25	0.9500	C68—H68	0.9500
			
C8—Sn1—C1	176.39 (13)	C26—C27—H27	120.0
C8—Sn1—N2	87.48 (10)	C28—C27—H27	120.0
C1—Sn1—N2	89.03 (10)	C27—C28—C23	120.0
C8—Sn1—N1	89.23 (10)	C27—C28—H28	120.0
C1—Sn1—N1	88.62 (12)	C23—C28—H28	120.0
N2—Sn1—N1	68.20 (8)	C30—C29—C34	120.0
C8—Sn1—Cl4	90.57 (8)	C30—C29—C22	120.59 (15)
C1—Sn1—Cl4	92.39 (9)	C34—C29—C22	118.93 (15)
N2—Sn1—Cl4	159.62 (6)	C31—C30—C29	120.0
N1—Sn1—Cl4	91.50 (6)	C31—C30—H30	120.0
C8—Sn1—Cl3	90.80 (9)	C29—C30—H30	120.0
C1—Sn1—Cl3	90.74 (11)	C30—C31—C32	120.0
N2—Sn1—Cl3	100.57 (6)	C30—C31—H31	120.0
N1—Sn1—Cl3	168.76 (6)	C32—C31—H31	120.0
Cl4—Sn1—Cl3	99.74 (3)	C33—C32—C31	120.0
C42—Sn2—C35	171.34 (10)	C33—C32—H32	120.0
C42—Sn2—N6	86.58 (9)	C31—C32—H32	120.0
C35—Sn2—N6	85.90 (9)	C32—C33—C34	120.0
C42—Sn2—N5	91.92 (9)	C32—C33—H33	120.0
C35—Sn2—N5	89.25 (9)	C34—C33—H33	120.0
N6—Sn2—N5	68.00 (7)	C33—C34—C29	120.0
C42—Sn2—Cl8	93.19 (8)	C33—C34—H34	120.0
C35—Sn2—Cl8	95.37 (8)	C29—C34—H34	120.0
N6—Sn2—Cl8	158.90 (6)	C36'—C35—Sn2	119.1 (4)
N5—Sn2—Cl8	90.93 (5)	C36—C35—Sn2	119.17 (19)
C42—Sn2—Cl7	88.67 (8)	C36'—C35—H35A	99.3
C35—Sn2—Cl7	88.54 (7)	C36—C35—H35A	107.5
N6—Sn2—Cl7	100.93 (5)	Sn2—C35—H35A	107.5
N5—Sn2—Cl7	168.85 (5)	C36'—C35—H35B	115.2
Cl8—Sn2—Cl7	100.15 (2)	C36—C35—H35B	107.5
C15—N1—C19	118.3 (3)	Sn2—C35—H35B	107.5
C15—N1—Sn1	123.8 (2)	H35A—C35—H35B	107.0
C19—N1—Sn1	117.81 (18)	C36'—C35—H35C	106.7
N3—N2—C20	120.0 (2)	C36—C35—H35C	114.3
N3—N2—Sn1	119.81 (18)	Sn2—C35—H35C	108.3
C20—N2—Sn1	119.86 (18)	C36'—C35—H35D	106.8
C21—N3—N2	118.7 (2)	C36—C35—H35D	98.5
C22—N4—C20	116.6 (2)	Sn2—C35—H35D	108.1
C49—N5—C53	118.2 (2)	H35C—C35—H35D	107.4
C49—N5—Sn2	123.98 (18)	C37—C36—C41	120.0
C53—N5—Sn2	117.69 (17)	C37—C36—C35	121.18 (18)
C54—N6—N7	120.1 (2)	C41—C36—C35	118.77 (18)
C54—N6—Sn2	118.63 (16)	C36—C37—C38	120.0
N7—N6—Sn2	121.17 (16)	C36—C37—Cl5	121.56 (14)
C56—N7—N6	118.2 (2)	C38—C37—Cl5	118.43 (14)
C55—N8—C54	116.6 (2)	C37—C38—C39	120.0
C2'—C1—Sn1	116.3 (6)	C37—C38—H38	120.0
C2—C1—Sn1	115.6 (6)	C39—C38—H38	120.0
C2'—C1—H1A	108.6	C40—C39—C38	120.0
C2—C1—H1A	108.4	C40—C39—H39	120.0
Sn1—C1—H1A	108.4	C38—C39—H39	120.0
C2'—C1—H1B	107.4	C41—C40—C39	120.0
C2—C1—H1B	108.4	C41—C40—H40	120.0
Sn1—C1—H1B	108.4	C39—C40—H40	120.0
H1A—C1—H1B	107.4	C40—C41—C36	120.0
C2'—C1—H1C	108.2	C40—C41—H41	120.0
C2—C1—H1C	108.0	C36—C41—H41	120.0
Sn1—C1—H1C	108.2	C37'—C36'—C41'	120.0
C2'—C1—H1D	108.2	C37'—C36'—C35	121.0 (5)
C2—C1—H1D	109.2	C41'—C36'—C35	118.9 (5)
Sn1—C1—H1D	108.2	C36'—C37'—C38'	120.0
H1C—C1—H1D	107.4	C36'—C37'—Cl5'	121.7 (4)
C3—C2—C7	120.0	C38'—C37'—Cl5'	118.3 (4)
C3—C2—C1	124.8 (5)	C39'—C38'—C37'	120.0
C7—C2—C1	115.1 (5)	C39'—C38'—H38'	120.0
C4—C3—C2	120.0	C37'—C38'—H38'	120.0
C4—C3—Cl1	118.5 (4)	C38'—C39'—C40'	120.0
C2—C3—Cl1	121.5 (4)	C38'—C39'—H39'	120.0
C3—C4—C5	120.0	C40'—C39'—H39'	120.0
C3—C4—H4	120.0	C39'—C40'—C41'	120.0
C5—C4—H4	120.0	C39'—C40'—H40'	120.0
C4—C5—C6	120.0	C41'—C40'—H40'	120.0
C4—C5—H5	120.0	C40'—C41'—C36'	120.0
C6—C5—H5	120.0	C40'—C41'—H41'	120.0
C5—C6—C7	120.0	C36'—C41'—H41'	120.0
C5—C6—H6	120.0	C43—C42—Sn2	114.53 (17)
C7—C6—H6	120.0	C43—C42—H42A	108.6
C6—C7—C2	120.0	Sn2—C42—H42A	108.6
C6—C7—H7	120.0	C43—C42—H42B	108.6
C2—C7—H7	120.0	Sn2—C42—H42B	108.6
C3'—C2'—C7'	120.0	H42A—C42—H42B	107.6
C3'—C2'—C1	115.2 (5)	C44—C43—C48	120.0
C7'—C2'—C1	124.7 (5)	C44—C43—C42	121.60 (16)
C2'—C3'—C4'	120.0	C48—C43—C42	117.96 (16)
C2'—C3'—Cl1'	121.8 (4)	C43—C44—C45	120.0
C4'—C3'—Cl1'	118.2 (4)	C43—C44—Cl6	122.75 (12)
C5'—C4'—C3'	120.0	C45—C44—Cl6	117.07 (11)
C5'—C4'—H4'	120.0	C46—C45—C44	120.0
C3'—C4'—H4'	120.0	C46—C45—H45	120.0
C4'—C5'—C6'	120.0	C44—C45—H45	120.0
C4'—C5'—H5'	120.0	C45—C46—C47	120.0
C6'—C5'—H5'	120.0	C45—C46—H46	120.0
C5'—C6'—C7'	120.0	C47—C46—H46	120.0
C5'—C6'—H6'	120.0	C48—C47—C46	120.0
C7'—C6'—H6'	120.0	C48—C47—H47	120.0
C6'—C7'—C2'	120.0	C46—C47—H47	120.0
C6'—C7'—H7'	120.0	C47—C48—C43	120.0
C2'—C7'—H7'	120.0	C47—C48—H48	120.0
C9—C8—Sn1	117.65 (18)	C43—C48—H48	120.0
C9—C8—H8A	107.9	N5—C49—C50	122.6 (3)
Sn1—C8—H8A	107.9	N5—C49—H49	118.7
C9—C8—H8B	107.9	C50—C49—H49	118.7
Sn1—C8—H8B	107.9	C51—C50—C49	119.0 (3)
H8A—C8—H8B	107.2	C51—C50—H50	120.5
C10—C9—C14	120.0	C49—C50—H50	120.5
C10—C9—C8	120.91 (17)	C50—C51—C52	119.2 (3)
C14—C9—C8	119.01 (17)	C50—C51—H51	120.4
C11—C10—C9	120.0	C52—C51—H51	120.4
C11—C10—Cl2	117.39 (11)	C53—C52—C51	118.2 (3)
C9—C10—Cl2	122.61 (11)	C53—C52—H52	120.9
C10—C11—C12	120.0	C51—C52—H52	120.9
C10—C11—H11	120.0	N5—C53—C52	122.9 (2)
C12—C11—H11	120.0	N5—C53—C54	116.5 (2)
C13—C12—C11	120.0	C52—C53—C54	120.6 (2)
C13—C12—H12	120.0	N6—C54—N8	124.5 (2)
C11—C12—H12	120.0	N6—C54—C53	117.5 (2)
C12—C13—C14	120.0	N8—C54—C53	118.0 (2)
C12—C13—H13	120.0	N8—C55—C56	119.4 (2)
C14—C13—H13	120.0	N8—C55—C57	115.3 (2)
C13—C14—C9	120.0	C56—C55—C57	125.3 (2)
C13—C14—H14	120.0	N7—C56—C55	120.9 (2)
C9—C14—H14	120.0	N7—C56—C63	114.9 (2)
N1—C15—C16	122.6 (3)	C55—C56—C63	124.2 (2)
N1—C15—H15	118.7	C58—C57—C62	120.0
C16—C15—H15	118.7	C58—C57—C55	118.65 (14)
C17—C16—C15	118.8 (3)	C62—C57—C55	120.94 (14)
C17—C16—H16	120.6	C59—C58—C57	120.0
C15—C16—H16	120.6	C59—C58—H58	120.0
C16—C17—C18	119.6 (3)	C57—C58—H58	120.0
C16—C17—H17	120.2	C58—C59—C60	120.0
C18—C17—H17	120.2	C58—C59—H59	120.0
C17—C18—C19	118.0 (3)	C60—C59—H59	120.0
C17—C18—H18	121.0	C61—C60—C59	120.0
C19—C18—H18	121.0	C61—C60—H60	120.0
N1—C19—C18	122.6 (3)	C59—C60—H60	120.0
N1—C19—C20	116.2 (3)	C60—C61—C62	120.0
C18—C19—C20	121.2 (3)	C60—C61—H61	120.0
N2—C20—N4	124.1 (3)	C62—C61—H61	120.0
N2—C20—C19	117.0 (2)	C61—C62—C57	120.0
N4—C20—C19	118.8 (3)	C61—C62—H62	120.0
N3—C21—C22	119.7 (2)	C57—C62—H62	120.0
N3—C21—C23	115.1 (2)	C64—C63—C68	120.0
C22—C21—C23	125.2 (2)	C64—C63—C56	121.38 (14)
N4—C22—C21	119.7 (3)	C68—C63—C56	118.44 (14)
N4—C22—C29	115.7 (2)	C65—C64—C63	120.0
C21—C22—C29	124.4 (2)	C65—C64—H64	120.0
C24—C23—C28	120.0	C63—C64—H64	120.0
C24—C23—C21	120.99 (17)	C66—C65—C64	120.0
C28—C23—C21	119.01 (17)	C66—C65—H65	120.0
C25—C24—C23	120.0	C64—C65—H65	120.0
C25—C24—H24	120.0	C65—C66—C67	120.0
C23—C24—H24	120.0	C65—C66—H66	120.0
C24—C25—C26	120.0	C67—C66—H66	120.0
C24—C25—H25	120.0	C68—C67—C66	120.0
C26—C25—H25	120.0	C68—C67—H67	120.0
C25—C26—C27	120.0	C66—C67—H67	120.0
C25—C26—H26	120.0	C67—C68—C63	120.0
C27—C26—H26	120.0	C67—C68—H68	120.0
C26—C27—C28	120.0	C63—C68—H68	120.0
			
C8—Sn1—N1—C15	−94.3 (2)	N3—C21—C23—C28	−38.4 (3)
C1—Sn1—N1—C15	88.6 (2)	C22—C21—C23—C28	141.9 (2)
N2—Sn1—N1—C15	178.1 (2)	C28—C23—C24—C25	0.0
Cl4—Sn1—N1—C15	−3.8 (2)	C21—C23—C24—C25	−179.4 (2)
Cl3—Sn1—N1—C15	175.4 (2)	C23—C24—C25—C26	0.0
C8—Sn1—N1—C19	87.1 (2)	C24—C25—C26—C27	0.0
C1—Sn1—N1—C19	−90.0 (2)	C25—C26—C27—C28	0.0
N2—Sn1—N1—C19	−0.52 (19)	C26—C27—C28—C23	0.0
Cl4—Sn1—N1—C19	177.6 (2)	C24—C23—C28—C27	0.0
Cl3—Sn1—N1—C19	−3.2 (5)	C21—C23—C28—C27	179.4 (2)
C8—Sn1—N2—N3	89.1 (2)	N4—C22—C29—C30	133.9 (2)
C1—Sn1—N2—N3	−91.8 (2)	C21—C22—C29—C30	−41.6 (3)
N1—Sn1—N2—N3	179.3 (2)	N4—C22—C29—C34	−38.1 (3)
Cl4—Sn1—N2—N3	173.92 (14)	C21—C22—C29—C34	146.4 (2)
Cl3—Sn1—N2—N3	−1.3 (2)	C34—C29—C30—C31	0.0
C8—Sn1—N2—C20	−83.9 (2)	C22—C29—C30—C31	−171.9 (2)
C1—Sn1—N2—C20	95.2 (2)	C29—C30—C31—C32	0.0
N1—Sn1—N2—C20	6.29 (19)	C30—C31—C32—C33	0.0
Cl4—Sn1—N2—C20	0.9 (3)	C31—C32—C33—C34	0.0
Cl3—Sn1—N2—C20	−174.24 (19)	C32—C33—C34—C29	0.0
C20—N2—N3—C21	5.5 (4)	C30—C29—C34—C33	0.0
Sn1—N2—N3—C21	−167.50 (19)	C22—C29—C34—C33	172.1 (2)
C42—Sn2—N5—C49	89.3 (2)	N6—Sn2—C35—C36'	58.9 (4)
C35—Sn2—N5—C49	−99.3 (2)	N5—Sn2—C35—C36'	−9.1 (4)
N6—Sn2—N5—C49	174.8 (2)	Cl8—Sn2—C35—C36'	−100.0 (4)
Cl8—Sn2—N5—C49	−3.9 (2)	Cl7—Sn2—C35—C36'	160.0 (4)
Cl7—Sn2—N5—C49	−177.8 (2)	N6—Sn2—C35—C36	48.0 (2)
C42—Sn2—N5—C53	−95.51 (19)	N5—Sn2—C35—C36	−20.0 (2)
C35—Sn2—N5—C53	75.90 (19)	Cl8—Sn2—C35—C36	−110.84 (19)
N6—Sn2—N5—C53	−9.98 (17)	Cl7—Sn2—C35—C36	149.10 (19)
Cl8—Sn2—N5—C53	171.26 (18)	C36'—C35—C36—C37	−6(3)
Cl7—Sn2—N5—C53	−2.7 (4)	Sn2—C35—C36—C37	86.3 (2)
C42—Sn2—N6—C54	104.8 (2)	C36'—C35—C36—C41	177 (3)
C35—Sn2—N6—C54	−79.5 (2)	Sn2—C35—C36—C41	−91.2 (2)
N5—Sn2—N6—C54	11.35 (18)	C41—C36—C37—C38	0.0
Cl8—Sn2—N6—C54	14.8 (3)	C35—C36—C37—C38	−177.4 (3)
Cl7—Sn2—N6—C54	−167.21 (18)	C41—C36—C37—Cl5	−178.96 (16)
C42—Sn2—N6—N7	−79.56 (19)	C35—C36—C37—Cl5	3.6 (2)
C35—Sn2—N6—N7	96.13 (19)	C36—C37—C38—C39	0.0
N5—Sn2—N6—N7	−173.0 (2)	Cl5—C37—C38—C39	178.99 (16)
Cl8—Sn2—N6—N7	−169.56 (12)	C37—C38—C39—C40	0.0
Cl7—Sn2—N6—N7	8.42 (18)	C38—C39—C40—C41	0.0
C54—N6—N7—C56	0.5 (3)	C39—C40—C41—C36	0.0
Sn2—N6—N7—C56	−175.11 (17)	C37—C36—C41—C40	0.0
N2—Sn1—C1—C2'	22.6 (4)	C35—C36—C41—C40	177.5 (3)
N1—Sn1—C1—C2'	90.8 (4)	C36—C35—C36'—C37'	−7(2)
Cl4—Sn1—C1—C2'	−177.8 (4)	Sn2—C35—C36'—C37'	−100.2 (6)
Cl3—Sn1—C1—C2'	−78.0 (4)	C36—C35—C36'—C41'	169 (3)
N2—Sn1—C1—C2	23.4 (4)	Sn2—C35—C36'—C41'	76.3 (4)
N1—Sn1—C1—C2	91.6 (4)	C41'—C36'—C37'—C38'	0.0
Cl4—Sn1—C1—C2	−176.9 (4)	C35—C36'—C37'—C38'	176.4 (9)
Cl3—Sn1—C1—C2	−77.2 (4)	C41'—C36'—C37'—Cl5'	179.4 (2)
C2'—C1—C2—C3	37 (69)	C35—C36'—C37'—Cl5'	−4.2 (8)
Sn1—C1—C2—C3	−95.9 (10)	C36'—C37'—C38'—C39'	0.0
C2'—C1—C2—C7	−146 (70)	Cl5'—C37'—C38'—C39'	−179.4 (2)
Sn1—C1—C2—C7	80.9 (4)	C37'—C38'—C39'—C40'	0.0
C7—C2—C3—C4	0.0	C38'—C39'—C40'—C41'	0.0
C1—C2—C3—C4	176.7 (13)	C39'—C40'—C41'—C36'	0.0
C7—C2—C3—Cl1	179.4 (2)	C37'—C36'—C41'—C40'	0.0
C1—C2—C3—Cl1	−3.9 (13)	C35—C36'—C41'—C40'	−176.5 (8)
C2—C3—C4—C5	0.0	N6—Sn2—C42—C43	−77.73 (18)
Cl1—C3—C4—C5	−179.4 (2)	N5—Sn2—C42—C43	−9.90 (18)
C3—C4—C5—C6	0.0	Cl8—Sn2—C42—C43	81.14 (17)
C4—C5—C6—C7	0.0	Cl7—Sn2—C42—C43	−178.76 (17)
C5—C6—C7—C2	0.0	Sn2—C42—C43—C44	94.20 (18)
C3—C2—C7—C6	0.0	Sn2—C42—C43—C48	−78.18 (19)
C1—C2—C7—C6	−177.0 (12)	C48—C43—C44—C45	0.0
C2—C1—C2'—C3'	−151 (71)	C42—C43—C44—C45	−172.2 (2)
Sn1—C1—C2'—C3'	−103.9 (8)	C48—C43—C44—Cl6	−174.94 (16)
C2—C1—C2'—C7'	25 (70)	C42—C43—C44—Cl6	12.8 (2)
Sn1—C1—C2'—C7'	72.3 (6)	C43—C44—C45—C46	0.0
C7'—C2'—C3'—C4'	0.0	Cl6—C44—C45—C46	175.22 (16)
C1—C2'—C3'—C4'	176.4 (12)	C44—C45—C46—C47	0.0
C7'—C2'—C3'—Cl1'	179.3 (2)	C45—C46—C47—C48	0.0
C1—C2'—C3'—Cl1'	−4.3 (11)	C46—C47—C48—C43	0.0
C2'—C3'—C4'—C5'	0.0	C44—C43—C48—C47	0.0
Cl1'—C3'—C4'—C5'	−179.4 (2)	C42—C43—C48—C47	172.5 (2)
C3'—C4'—C5'—C6'	0.0	C53—N5—C49—C50	−0.4 (4)
C4'—C5'—C6'—C7'	0.0	Sn2—N5—C49—C50	174.8 (2)
C5'—C6'—C7'—C2'	0.0	N5—C49—C50—C51	−0.8 (4)
C3'—C2'—C7'—C6'	0.0	C49—C50—C51—C52	0.9 (5)
C1—C2'—C7'—C6'	−176.0 (13)	C50—C51—C52—C53	0.0 (4)
N2—Sn1—C8—C9	−24.3 (2)	C49—N5—C53—C52	1.3 (4)
N1—Sn1—C8—C9	−92.5 (2)	Sn2—N5—C53—C52	−174.1 (2)
Cl4—Sn1—C8—C9	176.0 (2)	C49—N5—C53—C54	−176.4 (2)
Cl3—Sn1—C8—C9	76.3 (2)	Sn2—N5—C53—C54	8.1 (3)
Sn1—C8—C9—C10	102.9 (2)	C51—C52—C53—N5	−1.1 (4)
Sn1—C8—C9—C14	−80.2 (2)	C51—C52—C53—C54	176.5 (3)
C14—C9—C10—C11	0.0	N7—N6—C54—N8	−5.3 (4)
C8—C9—C10—C11	176.8 (2)	Sn2—N6—C54—N8	170.41 (19)
C14—C9—C10—Cl2	−179.96 (16)	N7—N6—C54—C53	172.6 (2)
C8—C9—C10—Cl2	−3.1 (2)	Sn2—N6—C54—C53	−11.7 (3)
C9—C10—C11—C12	0.0	C55—N8—C54—N6	4.6 (4)
Cl2—C10—C11—C12	179.96 (16)	C55—N8—C54—C53	−173.3 (2)
C10—C11—C12—C13	0.0	N5—C53—C54—N6	2.3 (3)
C11—C12—C13—C14	0.0	C52—C53—C54—N6	−175.5 (3)
C12—C13—C14—C9	0.0	N5—C53—C54—N8	−179.7 (2)
C10—C9—C14—C13	0.0	C52—C53—C54—N8	2.5 (4)
C8—C9—C14—C13	−176.9 (2)	C54—N8—C55—C56	0.5 (3)
C19—N1—C15—C16	2.7 (4)	C54—N8—C55—C57	179.9 (2)
Sn1—N1—C15—C16	−175.9 (2)	N6—N7—C56—C55	4.4 (4)
N1—C15—C16—C17	−1.1 (5)	N6—N7—C56—C63	−174.47 (19)
C15—C16—C17—C18	−1.0 (5)	N8—C55—C56—N7	−5.0 (4)
C16—C17—C18—C19	1.4 (5)	C57—C55—C56—N7	175.7 (2)
C15—N1—C19—C18	−2.2 (4)	N8—C55—C56—C63	173.8 (2)
Sn1—N1—C19—C18	176.5 (2)	C57—C55—C56—C63	−5.5 (4)
C15—N1—C19—C20	176.8 (2)	N8—C55—C57—C58	46.9 (3)
Sn1—N1—C19—C20	−4.5 (3)	C56—C55—C57—C58	−133.8 (2)
C17—C18—C19—N1	0.2 (5)	N8—C55—C57—C62	−125.8 (2)
C17—C18—C19—C20	−178.8 (3)	C56—C55—C57—C62	53.5 (3)
N3—N2—C20—N4	−8.8 (4)	C62—C57—C58—C59	0.0
Sn1—N2—C20—N4	164.2 (2)	C55—C57—C58—C59	−172.76 (19)
N3—N2—C20—C19	176.0 (2)	C57—C58—C59—C60	0.0
Sn1—N2—C20—C19	−11.0 (3)	C58—C59—C60—C61	0.0
C22—N4—C20—N2	1.4 (4)	C59—C60—C61—C62	0.0
C22—N4—C20—C19	176.5 (2)	C60—C61—C62—C57	0.0
N1—C19—C20—N2	10.2 (4)	C58—C57—C62—C61	0.0
C18—C19—C20—N2	−170.8 (3)	C55—C57—C62—C61	172.59 (19)
N1—C19—C20—N4	−165.3 (3)	N7—C56—C63—C64	−125.0 (2)
C18—C19—C20—N4	13.7 (4)	C55—C56—C63—C64	56.1 (3)
N2—N3—C21—C22	4.2 (4)	N7—C56—C63—C68	59.9 (2)
N2—N3—C21—C23	−175.5 (2)	C55—C56—C63—C68	−119.0 (2)
C20—N4—C22—C21	8.4 (4)	C68—C63—C64—C65	0.0
C20—N4—C22—C29	−167.3 (2)	C56—C63—C64—C65	−175.05 (19)
N3—C21—C22—N4	−11.5 (4)	C63—C64—C65—C66	0.0
C23—C21—C22—N4	168.2 (2)	C64—C65—C66—C67	0.0
N3—C21—C22—C29	163.9 (2)	C65—C66—C67—C68	0.0
C23—C21—C22—C29	−16.5 (4)	C66—C67—C68—C63	0.0
N3—C21—C23—C24	141.0 (2)	C64—C63—C68—C67	0.0
C22—C21—C23—C24	−38.7 (3)	C56—C63—C68—C67	175.19 (19)
